# What Synthesis Methodology Should I Use? A Review and Analysis of Approaches to Research Synthesis

**DOI:** 10.3934/publichealth.2016.1.172

**Published:** 2016-03-30

**Authors:** Kara Schick-Makaroff, Marjorie MacDonald, Marilyn Plummer, Judy Burgess, Wendy Neander

**Affiliations:** 1 Faculty of Nursing, University of Alberta, Edmonton, AB, Canada; 2 School of Nursing, University of Victoria, Victoria, BC, Canada; 3 College of Nursing, Camosun College, Victoria, BC, Canada; 4 Student Services, University Health Services, Victoria, BC, Canada

**Keywords:** research synthesis, systematic review, knowledge synthesis, methodology

## Abstract

**Background:**

When we began this process, we were doctoral students and a faculty member in a research methods course. As students, we were facing a review of the literature for our dissertations. We encountered several different ways of conducting a review but were unable to locate any resources that synthesized all of the various synthesis methodologies. Our purpose is to present a comprehensive overview and assessment of the main approaches to research synthesis. We use ‘research synthesis’ as a broad overarching term to describe various approaches to combining, integrating, and synthesizing research findings.

**Methods:**

We conducted an integrative review of the literature to explore the historical, contextual, and evolving nature of research synthesis. We searched five databases, reviewed websites of key organizations, hand-searched several journals, and examined relevant texts from the reference lists of the documents we had already obtained.

**Results:**

We identified four broad categories of research synthesis methodology including conventional, quantitative, qualitative, and emerging syntheses. Each of the broad categories was compared to the others on the following: key characteristics, purpose, method, product, context, underlying assumptions, unit of analysis, strengths and limitations, and when to use each approach.

**Conclusions:**

The current state of research synthesis reflects significant advancements in emerging synthesis studies that integrate diverse data types and sources. New approaches to research synthesis provide a much broader range of review alternatives available to health and social science students and researchers.

## 1. Introduction

Since the turn of the century, public health emergencies have been identified worldwide, particularly related to infectious diseases. For example, the Severe Acute Respiratory Syndrome (SARS) epidemic in Canada in 2002-2003, the recent Ebola epidemic in Africa, and the ongoing HIV/AIDs pandemic are global health concerns. There have also been dramatic increases in the prevalence of chronic diseases around the world [1]–[3]. These epidemiological challenges have raised concerns about the ability of health systems worldwide to address these crises. As a result, public health systems reform has been initiated in a number of countries. In Canada, as in other countries, the role of evidence to support public health reform and improve population health has been given high priority. Yet, there continues to be a significant gap between the production of evidence through research and its application in practice [4]–[5]. One strategy to address this gap has been the development of new research synthesis methodologies to deal with the time-sensitive and wide ranging evidence needs of policy makers and practitioners in all areas of health care, including public health.

As doctoral nursing students facing a review of the literature for our dissertations, and as a faculty member teaching a research methods course, we encountered several ways of conducting a research synthesis but found no comprehensive resources that discussed, compared, and contrasted various synthesis methodologies on their purposes, processes, strengths and limitations. To complicate matters, writers use terms interchangeably or use different terms to mean the same thing, and the literature is often contradictory about various approaches. Some texts [6],[7]–[9] did provide a preliminary understanding about how research synthesis had been taken up in nursing, but these did not meet our requirements. Thus, in this article we address the need for a comprehensive overview of research synthesis methodologies to guide public health, health care, and social science researchers and practitioners.

Research synthesis is relatively new in public health but has a long history in other fields dating back to the late 1800s. Research synthesis, a research process in its own right [10], has become more prominent in the wake of the evidence-based movement of the 1990s. Research syntheses have found their advocates and detractors in all disciplines, with challenges to the processes of systematic review and meta-analysis, in particular, being raised by critics of evidence-based healthcare [11]–[13].

## 2. Purpose

Our purpose was to conduct an integrative review of the literature to explore the historical, contextual, and evolving nature of research synthesis [14]–[15]. We synthesize and critique the main approaches to research synthesis that are relevant for public health, health care, and social scientists. *Research synthesis* is the overarching term we use to describe approaches to combining, aggregating, integrating, and synthesizing primary research findings. Each synthesis methodology draws on different types of findings depending on the purpose and product of the chosen synthesis (see Additional File 1).

## 3. Method of Review

Based on our current knowledge of the literature, we identified these approaches to include in our review: systematic review, meta-analysis, qualitative meta-synthesis, meta-narrative synthesis, scoping review, rapid review, realist synthesis, concept analysis, literature review, and integrative review. Our first step was to divide the synthesis types among the research team. Each member did a preliminary search to identify key texts. The team then met to develop search terms and a framework to guide the review.

Over the period of 2008 to 2012 we extensively searched the literature, updating our search at several time points, not restricting our search by date. The dates of texts reviewed range from 1967 to 2015. We used the terms above combined with the term “method* (e.g., “realist synthesis” and “method*) in the database *Health Source: Academic Edition* (includes Medline and CINAHL). This search yielded very few texts on some methodologies and many on others. We realized that many documents on research synthesis had not been picked up in the search. Therefore, we also searched Google Scholar, PubMed, ERIC, and Social Science Index, as well as the websites of key organizations such as the Joanna Briggs Institute, the University of York Centre for Evidence-Based Nursing, and the Cochrane Collaboration database. We hand searched several nursing, social science, public health and health policy journals. Finally, we traced relevant documents from the references in obtained texts.

We included works that met the following inclusion criteria: (1) published in English; (2) discussed the history of research synthesis; (3) explicitly described the approach and specific methods; or (4) identified issues, challenges, strengths and limitations of the particular methodology. We excluded research reports that resulted from the use of particular synthesis methodologies unless they also included criteria 2, 3, or 4 above.

Based on our search, we identified additional types of research synthesis (e.g., meta-interpretation, best evidence synthesis, critical interpretive synthesis, meta-summary, grounded formal theory). Still, we missed some important developments in meta-analysis, for example, identified by the journal's reviewers that have now been discussed briefly in the paper. The final set of 197 texts included in our review comprised theoretical, empirical, and conceptual papers, books, editorials and commentaries, and policy documents.

In our preliminary review of key texts, the team inductively developed a framework of the important elements of each method for comparison. In the next phase, each text was read carefully, and data for these elements were extracted into a table for comparison on the points of: key characteristics, purpose, methods, and product; see Additional File 1). Once the data were grouped and extracted, we synthesized across categories based on the following additional points of comparison: complexity of the process, degree of systematization, consideration of context, underlying assumptions, unit of analysis, and when to use each approach. In our results, we discuss our comparison of the various synthesis approaches on the elements above. Drawing only on documents for the review, ethics approval was not required.

## 4. Results

We identified four broad categories of research synthesis methodology: Conventional, quantitative, qualitative, and emerging syntheses. From our dataset of 197 texts, we had 14 texts on conventional synthesis, 64 on quantitative synthesis, 78 on qualitative synthesis, and 41 on emerging syntheses. Table 1 provides an overview of the four types of research synthesis, definitions, types of data used, products, and examples of the methodology.

Although we group these types of synthesis into four broad categories on the basis of similarities, each type within a category has unique characteristics, which may differ from the overall group similarities. Each could be explored in greater depth to tease out their unique characteristics, but detailed comparison is beyond the scope of this article.

Additional File 1 presents one or more selected types of synthesis that represent the broad category but is not an exhaustive presentation of all types within each category. It provides more depth for specific examples from each category of synthesis on the characteristics, purpose, methods, and products than is found in Table 1.

### 4.1 Key Characteristics

#### 4.1.1 What is it?

Here we draw on two types of categorization. First, we utilize Dixon Woods et al.'s [49] classification of research syntheses as being either *integrative* or *interpretive*. (Please note that integrative syntheses are not the same as an integrative review as defined in Additional File 1.) Second, we use Popay's [80] *enhancement* and *epistemological models*.

The defining characteristics of *integrative* syntheses are that they involve summarizing the data achieved by pooling data [49]. Integrative syntheses include systematic reviews, meta-analyses, as well as scoping and rapid reviews because each of these focus on summarizing data. They also define concepts from the outset (although this may not always be true in scoping or rapid reviews) and deal with a well-specified phenomenon of interest.

*Interpretive* syntheses are primarily concerned with the development of concepts and theories that integrate concepts [49]. The analysis in interpretive synthesis is conceptual both in process and outcome, and “the product is not aggregations of data, but theory” [49], [p.12]. Interpretive syntheses involve induction and interpretation, and are primarily conceptual in process and outcome. Examples include integrative reviews, some systematic reviews, all of the qualitative syntheses, meta-narrative, realist and critical interpretive syntheses. Of note, both quantitative and qualitative studies can be either integrative or interpretive

The second categorization, *enhancement* versus *epistemological*, applies to those approaches that use multiple data types and sources [80]. Popay's [80] classification reflects the ways that qualitative data are valued in relation to quantitative data.

In the *enhancement model*, qualitative data adds something to quantitative analysis. The enhancement model is reflected in systematic reviews and meta-analyses that use some qualitative data to enhance interpretation and explanation. It may also be reflected in some rapid reviews that draw on quantitative data but use some qualitative data.

The *epistemological model* assumes that quantitative and qualitative data are equal and each has something unique to contribute. All of the other review approaches, except pure quantitative or qualitative syntheses, reflect the epistemological model because they value all data types equally but see them as contributing different understandings.

**Table 1.** Categories of Research Synthesis Methodology.

Types of Research Synthesis	Definition	Data Types Used	Products	Examples
1. Conventional Synthesis	Older forms of review with less-systematic examination, critique, and synthesis of the literature on a mature topic for re-conceptulization or on a new topic for preliminary conceptualization	Quantitative studies Qualitative studies Other types of data e.g., theoretical literature, policy	Narrative expression and summary Tables, charts, graphical displays, diagrams and maps Theory, theoretical/conceptual frameworks, or conceptual maps	Integrative review [14],[18]–[21] Narrative synthesis [16] Conventional literature review[17]
2. Quantitative Synthesis	Combining, aggregating, or integrating quantitative empirical research with data expressed in numeric form	Quantitative studies	Narrative expression and summary Mathematical scores Statements of generalizability	Systematic review [13],[26]–[27] Meta-analysis [22]–[25] Best evidence synthesis [28]–[30]
3. Qualitative Synthesis	Combining, aggregating, or integrating qualitative empirical research and/or theoretical work expressed in narrative form	Qualitative studies Other types of data e.g., theoretical literature	Narrative expression and summary Theory, theoretical/conceptual frameworks, or conceptual maps A definition	Meta-synthesis [31]–[44] Concept analysis [45]–[47] Grounded formal theory [37],[48]–[52] Meta-study [31],[37],[48]–[50],[53]–[54] Meta-analysis [37]–[38],[55]–[58] Meta-interpretation [59] Meta-ethnography [49]–[50],[60]–[62]
4. Emerging Synthesis	Newer syntheses that provide a systematic approach to synthesizing varied literature in a topic area that includes diverse data types	Quantitative studies Qualitative studies Other types of data e.g., theoretical work, grey literature, editorials, commentaries, policy, evaluations	Narrative expression and summary Tables, charts, graphical displays, diagrams and maps Mathematical scores Theory, theoretical/conceptual frameworks, or conceptual maps A report written for decision-makers	Scoping review [63] Rapid review [64]–[65] Rapid realist review [66] Meta-narrative synthesis [67]–[68] Realist synthesis [68]–[69] Meta-summary [70] Critical interpretive synthesis [71]–[74] Other types of mixed-research synthesis [49],[72],[75]–[79]

#### 4.1.2 Data type

By and large, the quantitative approaches (quantitative systematic review and meta-analysis) have typically used purely quantitative data (i.e., expressed in numeric form). More recently, both Cochrane [81] and Campbell [82] collaborations are grappling with the need to, and the process of, integrating qualitative research into a systematic review. The qualitative approaches use qualitative data (i.e., expressed in words). All of the emerging synthesis types, as well as the conventional integrative review, incorporate qualitative and quantitative study designs and data.

#### 4.1.3 Research question

Four types of research questions direct inquiry across the different types of syntheses. The first is a well-developed research question that gives direction to the synthesis (e.g., meta-analysis, systematic review, meta-study, concept analysis, rapid review, realist synthesis). The second begins as a broad general question that evolves and becomes more refined over the course of the synthesis (e.g., meta-ethnography, scoping review, meta-narrative, critical interpretive synthesis). In the third type, the synthesis begins with a phenomenon of interest and the question emerges in the analytic process (e.g., grounded formal theory). Lastly, there is no clear question, but rather a general review purpose (e.g., integrative review). Thus, the requirement for a well-defined question cuts across at least three of the synthesis types (e.g., quantitative, qualitative, and emerging).

#### 4.1.4 Quality appraisal

This is a contested issue within and between the four synthesis categories. There are strong proponents of quality appraisal in the quantitative traditions of systematic review and meta-analysis based on the need for strong studies that will not jeopardize validity of the overall findings. Nonetheless, there is no consensus on pre-defined criteria; many scales exist that vary dramatically in composition. This has methodological implications for the credibility of findings [83].

Specific methodologies from the conventional, qualitative, and emerging categories support quality appraisal but do so with caveats. In conventional integrative reviews appraisal is recommended, but depends on the sampling frame used in the study [18]. In meta-study, appraisal criteria are explicit but quality criteria are used in different ways depending on the specific requirements of the inquiry [54]. Among the emerging syntheses, meta-narrative review developers support appraisal of a study based on criteria from the research tradition of the primary study [67],[84]–[85]. Realist synthesis similarly supports the use of high quality evidence, but appraisal checklists are viewed with scepticism and evidence is judged based on relevance to the research question and whether a credible inference may be drawn [69]. Like realist, critical interpretive syntheses do not judge quality using standardized appraisal instruments. They will exclude fatally flawed studies, but there is no consensus on what ‘fatally flawed’ means [49],[71]. Appraisal is based on relevance to the inquiry, not rigor of the study.

There is no agreement on quality appraisal among qualitative meta-ethnographers with some supporting and others refuting the need for appraisal. [60],[62]. Opponents of quality appraisal are found among authors of qualitative (grounded formal theory and concept analysis) and emerging syntheses (scoping and rapid reviews) because quality is not deemed relevant to the intention of the synthesis; the studies being reviewed are not effectiveness studies where quality is extremely important. These qualitative synthesis are often reviews of theoretical developments where the concept itself is what is important, or reviews that provide quotations from the raw data so readers can make their own judgements about the relevance and utility of the data. For example, in formal grounded theory, the purpose of theory generation and authenticity of data used to generate the theory is not as important as the conceptual category. Inaccuracies may be corrected in other ways, such as using the constant comparative method, which facilitates development of theoretical concepts that are repeatedly found in the data [86]–[87]. For pragmatic reasons, evidence is not assessed in rapid and scoping reviews, in part to produce a timely product. The issue of quality appraisal is unresolved across the terrain of research synthesis and we consider this further in our discussion.

### 4.2 Purpose

All research syntheses share a common purpose -- to summarize, synthesize, or integrate research findings from diverse studies. This helps readers stay abreast of the burgeoning literature in a field. Our discussion here is at the level of the four categories of synthesis. Beginning with conventional literature syntheses, the overall purpose is to attend to mature topics for the purpose of re-conceptualization or to new topics requiring preliminary conceptualization [14]. Such syntheses may be helpful to consider contradictory evidence, map shifting trends in the study of a phenomenon, and describe the emergence of research in diverse fields [14]. The purpose here is to set the stage for a study by identifying what has been done, gaps in the literature, important research questions, or to develop a conceptual framework to guide data collection and analysis.

The purpose of quantitative systematic reviews is to combine, aggregate, or integrate empirical research to be able to generalize from a group of studies and determine the limits of generalization [27]. The focus of quantitative systematic reviews has been primarily on aggregating the results of studies evaluating the effectiveness of interventions using experimental, quasi-experimental, and more recently, observational designs. Systematic reviews can be done with or without quantitative meta-analysis but a meta-analysis always takes place within the context of a systematic review. Researchers must consider the review's purpose and the nature of their data in undertaking a quantitative synthesis; this will assist in determining the approach.

The purpose of qualitative syntheses is broadly to synthesize complex health experiences, practices, or concepts arising in healthcare environments. There may be various purposes depending on the qualitative methodology. For example, in hermeneutic studies the aim may be holistic explanation or understanding of a phenomenon [42], which is deepened by integrating the findings from multiple studies. In grounded formal theory, the aim is to produce a conceptual framework or theory expected to be applicable beyond the original study. Although not able to generalize from qualitative research in the statistical sense [88], qualitative researchers usually do want to say something about the applicability of their synthesis to other settings or phenomena. This notion of ‘theoretical generalization’ has been referred to as ‘transferability’ [89]–[90] and is an important criterion of rigour in qualitative research. It applies equally to the products of a qualitative synthesis in which the synthesis of multiple studies on the same phenomenon strengthens the ability to draw transferable conclusions.

The overarching purpose of emerging syntheses is challenging the more traditional types of syntheses, in part by using data from both quantitative and qualitative studies with diverse designs for analysis. Beyond this, however, each emerging synthesis methodology has a unique purpose. In meta-narrative review, the purpose is to identify different research traditions in the area, synthesize a complex and diverse body of research. Critical interpretive synthesis shares this characteristic. Although a distinctive approach, critical interpretive synthesis utilizes a modification of the analytic strategies of meta-ethnography [61] (e.g., reciprocal translational analysis, refutational synthesis, and lines of argument synthesis) but goes beyond the use of these to bring a critical perspective to bear in challenging the normative or epistemological assumptions in the primary literature [72]–[73]. The unique purpose of a realist synthesis is to amalgamate complex empirical evidence and theoretical understandings within a diverse body of literature to uncover the operative mechanisms and contexts that affect the outcomes of social interventions. In a scoping review, the intention is to find key concepts, examine the range of research in an area, and identify gaps in the literature. The purpose of a rapid review is comparable to that of a scoping review, but done quickly to meet the time-sensitive information needs of policy makers.

### 4.3 Method

#### 4.3.1 Degree of systematization

There are varying degrees of systematization across the categories of research synthesis. The most systematized are quantitative systematic reviews and meta-analyses. There are clear processes in each with judgments to be made at each step, although there are no agreed upon guidelines for this. The process is inherently subjective despite attempts to develop objective and systematic processes [91]–[92]. Mullen and Ramirez [27] suggest that there is often a false sense of rigour implied by the terms ‘systematic review’ and ‘meta-analysis’ because of their clearly defined procedures.

In comparison with some types of qualitative synthesis, concept analysis is quite procedural. Qualitative meta-synthesis also has defined procedures and is systematic, yet perhaps less so than concept analysis. Qualitative meta-synthesis starts in an unsystematic way but becomes more systematic as it unfolds. Procedures and frameworks exist for some of the emerging types of synthesis [e.g.,[50],[63],[71],[93]] but are not linear, have considerable flexibility, and are often messy with emergent processes [85]. Conventional literature reviews tend not to be as systematic as the other three types. In fact, the lack of systematization in conventional literature synthesis was the reason for the development of more systematic quantitative [17],[20] and qualitative [45]–[46],[61] approaches. Some authors in the field [18] have clarified processes for integrative reviews making them more systematic and rigorous, but most conventional syntheses remain relatively unsystematic in comparison with other types.

#### 4.3.2 Complexity of the process

Some synthesis processes are considerably more complex than others. Methodologies with clearly defined steps are arguably less complex than the more flexible and emergent ones. We know that any study encounters challenges and it is rare that a pre-determined research protocol can be followed exactly as intended. Not even the rigorous methods associated with Cochrane [81] systematic reviews and meta-analyses are always implemented exactly as intended. Even when dealing with numbers rather than words, interpretation is always part of the process. Our collective experience suggests that new methodologies (e.g., meta-narrative synthesis and realist synthesis) that integrate different data types and methods are more complex than conventional reviews or the rapid and scoping reviews.

### 4.4 Product

The products of research syntheses usually take three distinct formats (see Table 1 and Additional File 1 for further details). The first representation is in tables, charts, graphical displays, diagrams and maps as seen in integrative, scoping and rapid reviews, meta-analyses, and critical interpretive syntheses. The second type of synthesis product is the use of mathematical scores. Summary statements of effectiveness are mathematically displayed in meta-analyses (as an effect size), systematic reviews, and rapid reviews (statistical significance).

The third synthesis product may be a theory or theoretical framework. A mid-range theory can be produced from formal grounded theory, meta-study, meta-ethnography, and realist synthesis. Theoretical/conceptual frameworks or conceptual maps may be created in meta-narrative and critical interpretive syntheses, and integrative reviews. Concepts for use within theories are produced in concept analysis. While these three product types span the categories of research synthesis, narrative description and summary is used to present the products resulting from all methodologies.

### 4.5 Consideration of context

There are diverse ways that context is considered in the four broad categories of synthesis. Context may be considered to the extent that it features within primary studies for the purpose of the review. Context may also be understood as an integral aspect of both the phenomenon under study and the synthesis methodology (e.g., realist synthesis). Quantitative systematic reviews and meta-analyses have typically been conducted on studies using experimental and quasi-experimental designs and more recently observational studies, which control for contextual features to allow for understanding of the ‘true’ effect of the intervention [94].

More recently, systematic reviews have included covariates or mediating variables (i.e., contextual factors) to help explain variability in the results across studies [27]. Context, however, is usually handled in the narrative discussion of findings rather than in the synthesis itself. This lack of attention to context has been one criticism leveled against systematic reviews and meta-analyses, which restrict the types of research designs that are considered [e.g.,[95]].

When conventional literature reviews incorporate studies that deal with context, there is a place for considering contextual influences on the intervention or phenomenon. Reviews of quantitative experimental studies tend to be devoid of contextual considerations since the original studies are similarly devoid, but context might figure prominently in a literature review that incorporates both quantitative and qualitative studies.

Qualitative syntheses have been conducted on the contextual features of a particular phenomenon [33]. Paterson et al. [54] advise researchers to attend to how context may have influenced the findings of particular primary studies. In qualitative analysis, contextual features may form categories by which the data can be compared and contrasted to facilitate interpretation. Because qualitative research is often conducted to understand a phenomenon as a whole, context may be a focus, although this varies with the qualitative methodology. At the same time, the findings in a qualitative synthesis are abstracted from the original reports and taken to a higher level of conceptualization, thus removing them from the original context.

Meta-narrative synthesis [67],[84], because it draws on diverse research traditions and methodologies, may incorporate context into the analysis and findings. There is not, however, an explicit step in the process that directs the analyst to consider context. Generally, the research question guiding the synthesis is an important factor in whether context will be a focus.

More recent iterations of concept analysis [47],[96]–[97] explicitly consider context reflecting the assumption that a concept's meaning is determined by its context. Morse [47] points out, however, that Wilson's [98] approach to concept analysis, and those based on Wilson [e.g., [45]], identify attributes that are devoid of context, while Rodgers' [96],[99] evolutionary method considers context (e.g., antecedents, consequences, and relationships to other concepts) in concept development.

Realist synthesis [69] considers context as integral to the study. It draws on a critical realist logic of inquiry grounded in the work of Bhaskar [100], who argues that empirical co-occurrence of events is insufficient for inferring causation. One must identify generative mechanisms whose properties are causal and, depending on the situation, may nor may not be activated [94]. Context interacts with program/intervention elements and thus cannot be differentiated from the phenomenon [69]. This approach synthesizes evidence on generative mechanisms and analyzes contextual features that activate them; the result feeds back into the context. The focus is on what works, for whom, under what conditions, why and how [68].

### 4.6 Underlying Philosophical and Theoretical Assumptions

When we began our review, we ‘assumed’ that the assumptions underlying synthesis methodologies would be a distinguishing characteristic of synthesis types, and that we could compare the various types on their assumptions, explicit or implicit. We found, however, that many authors did not explicate the underlying assumptions of their methodologies, and it was difficult to infer them. Kirkevold [101] has argued that integrative reviews need to be carried out from an explicit philosophical or theoretical perspective. We argue this should be true for all types of synthesis.

Authors of some emerging synthesis approaches have been very explicit about their assumptions and philosophical underpinnings. An implicit assumption of most emerging synthesis methodologies is that quantitative systematic reviews and meta-analyses have limited utility in some fields [e.g., in public health –[13],[102]] and for some kinds of review questions like those about feasibility and appropriateness versus effectiveness [103]–[104]. They also assume that ontologically and epistemologically, both kinds of data can be combined. This is a significant debate in the literature because it is about the commensurability of overarching paradigms [105] but this is beyond the scope of this review.

Realist synthesis is philosophically grounded in critical realism or, as noted above, a realist logic of inquiry [93],[99],[106]–[107]. Key assumptions regarding the nature of interventions that inform critical realism have been described above in the section on context. See Pawson et al. [106] for more information on critical realism, the philosophical basis of realist synthesis.

Meta-narrative synthesis is explicitly rooted in a constructivist philosophy of science [108] in which knowledge is socially constructed rather than discovered, and what we take to be ‘truth’ is a matter of perspective. Reality has a pluralistic and plastic character, and there is no pre-existing ‘real world’ independent of human construction and language [109]. See Greenhalgh et al. [67],[85] and Greenhalgh & Wong [97] for more discussion of the constructivist basis of meta-narrative synthesis.

In the case of purely quantitative or qualitative syntheses, it may be an easier matter to uncover unstated assumptions because they are likely to be shared with those of the primary studies in the genre. For example, grounded formal theory shares the philosophical and theoretical underpinnings of grounded theory, rooted in the theoretical perspective of symbolic interactionism [110]–[111] and the philosophy of pragmatism [87],[112]–[114].

As with meta-narrative synthesis, meta-study developers identify constructivism as their interpretive philosophical foundation [54],[88]. Epistemologically, constructivism focuses on how people construct and re-construct knowledge about a specific phenomenon, and has three main assumptions: (1) reality is seen as multiple, at times even incompatible with the phenomenon under consideration; (2) just as primary researchers construct interpretations from participants' data, meta-study researchers also construct understandings about the primary researchers' original findings. Thus, meta-synthesis is a construction of a construction, or a meta-construction; and (3) all constructions are shaped by the historical, social and ideological context in which they originated [54]. The key message here is that reports of any synthesis would benefit from an explicit identification of the underlying philosophical perspectives to facilitate a better understanding of the results, how they were derived, and how they are being interpreted.

### 4.7 Unit of Analysis

The unit of analysis for each category of review is generally distinct. For the emerging synthesis approaches, the unit of analysis is specific to the intention. In meta-narrative synthesis it is the storyline in diverse research traditions; in rapid review or scoping review, it depends on the focus but could be a concept; and in realist synthesis, it is the theories rather than programs that are the units of analysis. The elements of theory that are important in the analysis are mechanisms of action, the context, and the outcome [107].

For qualitative synthesis, the units of analysis are generally themes, concepts or theories, although in meta-study, the units of analysis can be research findings (“meta-data-analysis”), research methods (“meta-method”) or philosophical/theoretical perspectives (“meta-theory”) [54]. In quantitative synthesis, the units of analysis range from specific statistics for systematic reviews to effect size of the intervention for meta-analysis. More recently, some systematic reviews focus on theories [115]–[116], therefore it depends on the research question. Similarly, within conventional literature synthesis the units of analysis also depend on the research purpose, focus and question as well as on the type of research methods incorporated into the review. What is important in all research syntheses, however, is that the unit of analysis needs to be made explicit. Unfortunately, this is not always the case.

### 4.8 Strengths and Limitations

In this section, we discuss the overarching strengths and limitations of synthesis methodologies as a whole and then highlight strengths and weaknesses across each of our four categories of synthesis.

#### 4.8.1 Strengths of Research Syntheses in General

With the vast proliferation of research reports and the increased ease of retrieval, research synthesis has become more accessible providing a way of looking broadly at the current state of research. The availability of syntheses helps researchers, practitioners, and policy makers keep up with the burgeoning literature in their fields without which evidence-informed policy or practice would be difficult. Syntheses explain variation and difference in the data helping us identify the relevance for our own situations; they identify gaps in the literature leading to new research questions and study designs. They help us to know when to replicate a study and when to avoid excessively duplicating research. Syntheses can inform policy and practice in a way that well-designed single studies cannot; they provide building blocks for theory that helps us to understand and explain our phenomena of interest.

#### 4.8.2 Limitations of Research Syntheses in General

The process of selecting, combining, integrating, and synthesizing across diverse study designs and data types can be complex and potentially rife with bias, even with those methodologies that have clearly defined steps. Just because a rigorous and standardized approach has been used does not mean that implicit judgements will not influence the interpretations and choices made at different stages.

In all types of synthesis, the quantity of data can be considerable, requiring difficult decisions about scope, which may affect relevance. The quantity of available data also has implications for the size of the research team. Few reviews these days can be done independently, in particular because decisions about inclusion and exclusion may require the involvement of more than one person to ensure reliability.

For all types of synthesis, it is likely that in areas with large, amorphous, and diverse bodies of literature, even the most sophisticated search strategies will not turn up all the relevant and important texts. This may be more important in some synthesis methodologies than in others, but the omission of key documents can influence the results of all syntheses. This issue can be addressed, at least in part, by including a library scientist on the research team as required by some funding agencies. Even then, it is possible to miss key texts. In this review, for example, because none of us are trained in or conduct meta-analyses, we were not even aware that we had missed some new developments in this field such as meta-regression [117]–[118], network meta-analysis [119]–[121], and the use of individual patient data in meta-analyses [122]–[123].

One limitation of systematic reviews and meta-analyses is that they rapidly go out of date. We thought this might be true for all types of synthesis, although we wondered if those that produce theory might not be somewhat more enduring. We have not answered this question but it is open for debate. For all types of synthesis, the analytic skills and the time required are considerable so it is clear that training is important before embarking on a review, and some types of review may not be appropriate for students or busy practitioners.

Finally, the quality of reporting in primary studies of all genres is variable so it is sometimes difficult to identify aspects of the study essential for the synthesis, or to determine whether the study meets quality criteria. There may be flaws in the original study, or journal page limitations may necessitate omitting important details. Reporting standards have been developed for some types of reviews (e.g., systematic review, meta-analysis, meta-narrative synthesis, realist synthesis); but there are no agreed upon standards for qualitative reviews. This is an important area for development in advancing the science of research synthesis.

#### 4.8.3 Strengths and Limitations of the Four Synthesis Types

The *conventional literature review* and now the increasingly common *integrative review* remain important and accessible approaches for students, practitioners, and experienced researchers who want to summarize literature in an area but do not have the expertise to use one of the more complex methodologies. Carefully executed, such reviews are very useful for synthesizing literature in preparation for research grants and practice projects. They can determine the state of knowledge in an area and identify important gaps in the literature to provide a clear rationale or theoretical framework for a study [14],[18]. There is a demand, however, for more rigour, with more attention to developing comprehensive search strategies and more systematic approaches to combining, integrating, and synthesizing the findings.

Generally, conventional reviews include diverse study designs and data types that facilitate comprehensiveness, which may be a strength on the one hand, but can also present challenges on the other. The complexity inherent in combining results from studies with diverse methodologies can result in bias and inaccuracies. The absence of clear guidelines about how to synthesize across diverse study types and data [18] has been a challenge for novice reviewers.

*Quantitative systematic reviews* and *meta-analyses* have been important in launching the field of evidence-based healthcare. They provide a systematic, orderly and auditable process for conducting a review and drawing conclusions [25]. They are arguably the most powerful approaches to understanding the effectiveness of healthcare interventions, especially when intervention studies on the same topic show very different results. When areas of research are dogged by controversy [25] or when study results go against strongly held beliefs, such approaches can reduce the uncertainty and bring strong evidence to bear on the controversy.

Despite their strengths, they also have limitations. Systematic reviews and meta-analyses do not provide a way of including complex literature comprising various types of evidence including qualitative studies, theoretical work, and epidemiological studies. Only certain types of design are considered and qualitative data are used in a limited way. This exclusion limits what can be learned in a topic area.

Meta-analyses are often not possible because of wide variability in study design, population, and interventions so they may have a narrow range of utility. New developments in meta-analysis, however, can be used to address some of these limitations. Network meta-analysis is used to explore relative efficacy of multiple interventions, even those that have never been compared in more conventional pairwise meta-analyses [121], allowing for improved clinical decision making [120]. The limitation is that network meta-analysis has only been used in medical/clinical applications [119] and not in public health. It has not yet been widely accepted and many methodological challenges remain [120]–[121]. Meta-regression is another development that combines meta-analytic and linear regression principles to address the fact that heterogeneity of results may compromise a meta-analysis [117]–[118]. The disadvantage is that many clinicians are unfamiliar with it and may incorrectly interpret results [117].

Some have accused meta-analysis of combining apples and oranges [124] raising questions in the field about their meaningfulness [25],[28]. More recently, the use of individual rather than aggregate data has been useful in facilitating greater comparability among studies [122]. In fact, Tomas et al. [123] argue that meta-analysis using individual data is now the gold standard although access to the raw data from other studies may be a challenge to obtain.

The usefulness of systematic reviews in synthesizing complex health and social interventions has also been challenged [102]. It is often difficult to synthesize their findings because such studies are “epistemologically diverse and methodologically complex” [[69], p.21]. Rigid inclusion/exclusion criteria may allow only experimental or quasi-experimental designs into consideration resulting in lost information that may well be useful to policy makers for tailoring an intervention to the context or understanding its acceptance by recipients.

*Qualitative syntheses* may be the type of review most fraught with controversy and challenge, while also bringing distinct strengths to the enterprise. Although these methodologies provide a comprehensive and systematic review approach, they do not generally provide definitive statements about intervention effectiveness. They do, however, address important questions about the development of theoretical concepts, patient experiences, acceptability of interventions, and an understanding about why interventions might work.

Most qualitative syntheses aim to produce a theoretically generalizable mid-range theory that explains variation across studies. This makes them more useful than single primary studies, which may not be applicable beyond the immediate setting or population. All provide a contextual richness that enhances relevance and understanding. Another benefit of some types of qualitative synthesis (e.g., grounded formal theory) is that the concept of saturation provides a sound rationale for limiting the number of texts to be included thus making reviews potentially more manageable. This contrasts with the requirements of systematic reviews and meta-analyses that require an exhaustive search.

Qualitative researchers debate about whether the findings of ontologically and epistemological diverse qualitative studies can actually be combined or synthesized [125] because methodological diversity raises many challenges for synthesizing findings. The products of different types of qualitative syntheses range from theory and conceptual frameworks, to themes and rich descriptive narratives. Can one combine the findings from a phenomenological study with the theory produced in a grounded theory study? Many argue yes, but many also argue no.

*Emerging synthesis* methodologies were developed to address some limitations inherent in other types of synthesis but also have their own issues. Because each type is so unique, it is difficult to identify overarching strengths of the entire category. An important strength, however, is that these newer forms of synthesis provide a systematic and rigorous approach to synthesizing a diverse literature base in a topic area that includes a range of data types such as: both quantitative and qualitative studies, theoretical work, case studies, evaluations, epidemiological studies, trials, and policy documents. More than conventional literature reviews and systematic reviews, these approaches provide explicit guidance on analytic methods for integrating different types of data. The assumption is that all forms of data have something to contribute to knowledge and theory in a topic area. All have a defined but flexible process in recognition that the methods may need to shift as knowledge develops through the process.

Many emerging synthesis types are helpful to policy makers and practitioners because they are usually involved as team members in the process to define the research questions, and interpret and disseminate the findings. In fact, engagement of stakeholders is built into the procedures of the methods. This is true for rapid reviews, meta-narrative syntheses, and realist syntheses. It is less likely to be the case for critical interpretive syntheses.

Another strength of some approaches (realist and meta-narrative syntheses) is that quality and publication standards have been developed to guide researchers, reviewers, and funders in judging the quality of the products [108],[126]–[127]. Training materials and online communities of practice have also been developed to guide users of realist and meta-narrative review methods [107],[128]. A unique strength of critical interpretive synthesis is that it takes a critical perspective on the process that may help reconceptualize the data in a way not considered by the primary researchers [72].

There are also challenges of these new approaches. The methods are new and there may be few published applications by researchers other than the developers of the methods, so new users often struggle with the application. The newness of the approaches means that there may not be mentors available to guide those unfamiliar with the methods. This is changing, however, and the number of applications in the literature is growing with publications by new users helping to develop the science of synthesis [e.g.,[129]]. However, the evolving nature of the approaches and their developmental stage present challenges for novice researchers.

### 4.9 When to Use Each Approach

Choosing an appropriate approach to synthesis will depend on the question you are asking, the purpose of the review, and the outcome or product you want to achieve. In Additional File 1, we discuss each of these to provide guidance to readers on making a choice about review type. If researchers want to know whether a particular type of intervention is effective in achieving its intended outcomes, then they might choose a quantitative systemic review with or without meta-analysis, possibly buttressed with qualitative studies to provide depth and explanation of the results. Alternately, if the concern is about whether an intervention is effective with different populations under diverse conditions in varying contexts, then a realist synthesis might be the most appropriate.

If researchers' concern is to develop theory, they might consider qualitative syntheses or some of the emerging syntheses that produce theory (e.g., critical interpretive synthesis, realist review, grounded formal theory, qualitative meta-synthesis). If the aim is to track the development and evolution of concepts, theories or ideas, or to determine how an issue or question is addressed across diverse research traditions, then meta-narrative synthesis would be most appropriate.

When the purpose is to review the literature in advance of undertaking a new project, particularly by graduate students, then perhaps an integrative review would be appropriate. Such efforts contribute towards the expansion of theory, identify gaps in the research, establish the rationale for studying particular phenomena, and provide a framework for interpreting results in ways that might be useful for influencing policy and practice.

For researchers keen to bring new insights, interpretations, and critical re-conceptualizations to a body of research, then qualitative or critical interpretive syntheses will provide an inductive product that may offer new understandings or challenges to the status quo. These can inform future theory development, or provide guidance for policy and practice.

## 5. Discussion

What is the current state of science regarding research synthesis? Public health, health care, and social science researchers or clinicians have previously used all four categories of research synthesis, and all offer a suitable array of approaches for inquiries. New developments in systematic reviews and meta-analysis are providing ways of addressing methodological challenges [117]–[123]. There has also been significant advancement in emerging synthesis methodologies and they are quickly gaining popularity. Qualitative meta-synthesis is still evolving, particularly given how new it is within the terrain of research synthesis. In the midst of this evolution, outstanding issues persist such as grappling with: the quantity of data, quality appraisal, and integration with knowledge translation. These topics have not been thoroughly addressed and need further debate.

### 5.1 Quantity of Data

We raise the question of whether it is possible or desirable to find all available studies for a synthesis that has this requirement (e.g., meta-analysis, systematic review, scoping, meta-narrative synthesis [25],[27],[63],[67],[84]–[85]). Is the synthesis of all available studies a realistic goal in light of the burgeoning literature? And how can this be sustained in the future, particularly as the emerging methodologies continue to develop and as the internet facilitates endless access? There has been surprisingly little discussion on this topic and the answers will have far-reaching implications for searching, sampling, and team formation.

Researchers and graduate students can no longer rely on their own independent literature search. They will likely need to ask librarians for assistance as they navigate multiple sources of literature and learn new search strategies. Although teams now collaborate with library scientists, syntheses are limited in that researchers must make decisions on the boundaries of the review, in turn influencing the study's significance. The size of a team may also be pragmatically determined to manage the search, extraction, and synthesis of the burgeoning data. There is no single answer to our question about the possibility or necessity of finding all available articles for a review. Multiple strategies that are situation specific are likely to be needed.

### 5.2 Quality Appraisal

While the issue of quality appraisal has received much attention in the synthesis literature, scholars are far from resolution. There may be no agreement about appraisal criteria in a given tradition. For example, the debate rages over the appropriateness of quality appraisal in qualitative synthesis where there are over 100 different sets of criteria and many do not overlap [49]. These differences may reflect disciplinary and methodological orientations, but diverse quality appraisal criteria may privilege particular types of research [49]. The decision to appraise is often grounded in ontological and epistemological assumptions. Nonetheless, diversity within and between categories of synthesis is likely to continue unless debate on the topic of quality appraisal continues and evolves toward consensus.

### 5.3 Integration with Knowledge Translation

If research syntheses are to make a difference to practice and ultimately to improve health outcomes, then we need to do a better job of knowledge translation. In the Canadian Institutes of Health Research (CIHR) definition of knowledge translation (KT), research or knowledge synthesis is an integral component [130]. Yet, with few exceptions [131]–[132], very little of the research synthesis literature even mentions the relationship of synthesis to KT nor does it discuss strategies to facilitate the integration of synthesis findings into policy and practice. The exception is in the emerging synthesis methodologies, some of which (e.g., realist and meta-narrative syntheses, scoping reviews) explicitly involve stakeholders or knowledge users. The argument is that engaging them in this way increases the likelihood that the knowledge generated will be translated into policy and practice. We suggest that a more explicit engagement with knowledge users in all types of synthesis would benefit the uptake of the research findings.

Research synthesis neither makes research more applicable to practice nor ensures implementation. Focus must now turn seriously towards translation of synthesis findings into knowledge products that are useful for health care practitioners in multiple areas of practice and develop appropriate strategies to facilitate their use. The burgeoning field of knowledge translation has, to some extent, taken up this challenge; however, the research-practice gap continues to plague us [133]–[134]. It is a particular problem for qualitative syntheses [131]. Although such syntheses have an important place in evidence-informed practice, little effort has gone into the challenge of translating the findings into useful products to guide practice [131].

### 5.4 Limitations

Our study took longer than would normally be expected for an integrative review. Each of us were primarily involved in our own dissertations or teaching/research positions, and so this study was conducted ‘off the sides of our desks.’ A limitation was that we searched the literature over the course of 4 years (from 2008–2012), necessitating multiple search updates. Further, we did not do a comprehensive search of the literature after 2012, thus the more recent synthesis literature was not systematically explored. We did, however, perform limited database searches from 2012–2015 to keep abreast of the latest methodological developments. Although we missed some new approaches to meta-analysis in our search, we did not find any new features of the synthesis methodologies covered in our review that would change the analysis or findings of this article. Lastly, we struggled with the labels used for the broad categories of research synthesis methodology because of our hesitancy to reinforce the divide between quantitative and qualitative approaches. However, it was very difficult to find alternative language that represented the types of data used in these methodologies. Despite our hesitancy in creating such an obvious divide, we were left with the challenge of trying to find a way of characterizing these broad types of syntheses.

## 6. Conclusion

Our findings offer methodological clarity for those wishing to learn about the broad terrain of research synthesis. We believe that our review makes transparent the issues and considerations in choosing from among the four broad categories of research synthesis. In summary, research synthesis has taken its place as a form of research in its own right. The methodological terrain has deep historical roots reaching back over the past 200 years, yet research synthesis remains relatively new to public health, health care, and social sciences in general. This is rapidly changing. New developments in systematic reviews and meta-analysis, and the emergence of new synthesis methodologies provide a vast array of options to review the literature for diverse purposes. New approaches to research synthesis and new analytic methods within existing approaches provide a much broader range of review alternatives for public health, health care, and social science students and researchers.

## Abbreviations

**Additional File 1:** Selected Types of Research Synthesis

Types of Research Synthesis	Key Characteristics	Purpose	Methods	Product
CONVENTIONAL **Integrative Review**	**What is it?** “The integrative literature review is a form of research that reviews, critiques, and synthesizes representative literature on a topic in an integrated way such that new frameworks and perspectives on the topic are generated” [[14], p.356]. **Data type:** Integrative literature reviews include studies using diverse methodologies (i.e., experimental and non-experimental research, as well as qualitative research) in order to more fully understand a phenomenon of interest. It may also include theoretical and empirical literature. **Research question:** Start by clearly identifying the problem that the review is addressing and the purpose of the review. There usually is not a specific research question, but rather a research purpose. **Quality appraisal:** The quality of primary sources may be appraised using broad criteria. How quality is evaluated will depend upon the sampling frame [18].	Integrative reviews are used to address mature topics in order to re-conceptualize the expanding and diverse literature on the topic. They are also used to comprehensively review new topics in need of preliminary conceptualization [14]. Integrative reviews should ultimately present the “state of the art” of knowledge, depict the breadth and depth of the topic, and contribute to greater understanding of the phenomenon [18].	Integrative reviews generally contain similar steps [14],[18], which include the following: Identify a clear problem. Determine the variables of interest (e.g., population, concept). State a specific research purpose. Define and clearly document a search strategy. Aim to locate as many of the existing studies as possible. Purposive sampling may be used along with a more comprehensive approach. Critically evaluate the quality of primary reviews depending on the sampling frame used in the integrative review. Identify a systematic analytic method. The constant comparative method [86],[135] is one overarching approach commonly used. Keep a record of the process of data analysis (e.g., hunches, decisions, ideas about interpretation). State methodological limitations.	Conclusions are often presented in a table/diagram. Explicit details from primary sources to support conclusions must be provided to demonstrate a logical chain of evidence. Torraco [14] suggests they can be represented in four forms: A research agenda, A taxonomy or conceptual classification of constructs, Alternative models/conceptual framework, and Metatheory. Results should emphasize implications for policy/practice [18].
QUANTITATIVE **Systematic Review (SR)**	**What is it?** A SR is a review of literature that uses systematic and explicit methods to identify, select, and critically appraise relevant research, and to collect and analyze data from the studies. Conducting a SR is analogous to conducting a primary study in that there are steps and protocols. It may or may not be done in conjunction with a meta-analysis. In Cochrane [81], a SR is identified as the highest form of evidence in support of interventions. By contrast, the Joanna Briggs Institute [104] does not define a SR as necessarily the highest form of evidence. As noted below, a meta-analysis is always a SR, but a SR is not always a meta-analysis. **Data type:** There is nothing that specifies data have to be quantitative, and the definition can apply to qualitative findings. Generally, however, the term has been used most frequently to apply to reviews of quantitative studies – traditional RCTs and experimental or quasi-experimental designs. More recently, both the Campbell and the Cochrane collaborations have been grappling with the need to, and the process of, integrating qualitative research into a SR. A number of studies have been published that do this [13],[75],[78],[135]–[138]. **Research question:** A well-defined research question is required. **Quality appraisal:** The Quality Appraisal section under MA above also applies to SR. Some researchers are developing standard reliable and valid quality appraisal tools to judge the quality of primary studies but there remains no consensus on which tools should be used. The Joanna Briggs Institute [104] has developed their own criteria to ensure that only the highest quality studies are included in SRs for nursing, but they hold that studies from any methodological position are relevant.	The purpose of a SR is to integrate empirical research for the purpose of generalizing from a group of studies. The reviewer is also seeking to discover the limits of generalization [27]. Often, the review focuses on questions of intervention effectiveness. Thus, the intent is to summarize across studies to obtain a summative judgment about the effectiveness of interventions. However, the Joanna Briggs Institute [104] suggests that for nursing, there is a concern not just with effectiveness but also with questions of appropriateness, meaningfulness and feasibility of health practices and delivery methods. Thus, SR's may have purposes other than to assess the effectiveness of interventions.	A number of authors have provided guidelines for conducting a SR [27] but they generally contain similar steps: Specify study aims and define research question. Set inclusion criteria for evidence. Design search strategy. Screen potential evidence against criteria for assessing quality. Design data collection protocol. Select appropriate metric to represent the magnitude of findings and assess likelihood they are due to chance. Code the primary studies. Analyze and display data using appropriate methods. Draw conclusions based on data. Discuss alternate interpretations in light of studies' strengths and limitations.	The products of a SR may include: A statement about the relative “effectiveness” of health care interventions, or about the appropriateness, feasibility, or meaningfulness of findings for particular purposes; A statement about the strength of the relationship between a particular intervention and specific outcomes. More recently, the product might be a statement about the convergence of theoretical perspectives on a topic. When done in conjunction with meta-analysis, the product is a mathematic score that represents the statements above.
QUANTITATIVE **Meta-Analysis****(M-A)**	**What is it?** M-A is the statistical analysis of a large collection of results from individual studies (usually interventions) for the purposes of integrating the findings, based on conversion to a common metric (effect size) to determine the overall effect and its magnitude. The term was coined by Gene Glass [22]–[23] but dates back to 1904 [17]. A M-A is always a SR (see above). **Data type:** Data are from quantitative research studies and findings, primarily randomized control trials. Increasingly there is use of experimental, quasi-experimental and some types of observational studies. Each primary study is abstracted and coded into a database. **Research question:** A clear, well-defined research question or hypothesis is required. **Quality appraisal:** Articles are usually appraised according to a set of pre-defined criteria but these criteria vary considerably and there are many methodological limitations [83]. Lower quality studies are not necessarily excluded and there is some debate about whether these should be included [24], [29]. When lower quality studies are included, the validity of the findings is often discussed in relation to the study quality.	Analytic M-As are conducted for the purpose of summarizing and integrating the results of individual primary studies to increase the power for detecting intervention effects, which may be small and insignificant in the individual studies [139]–[140]. Exploratory M-As are conducted to resolve controversy in a field or to pose and answer new questions. The main concern is to explain the variation in effect sizes.	Specific steps include [25]: Define the dependent and independent variables of interest. Collect the studies in a systematic way attempting to find all published and unpublished studies. Read methods carefully and if effect sizes are not reported, identify articles for information to calculate these. Examine variability among the obtained effect sizes informally with graphs and charts, to identify the possibility that moderator variables may account for the variability. Combine effects using several measures of their central tendency and explore reasons for differences if found. Examine the significance level of the indices of central tendency, usually employing confidence intervals around unweighted mean effect sizes in a random effects model. Using an examination of the binomial effect size display, evaluate the importance of the obtained effect size.	The product for M-A includes a narrative summary of the findings with a conclusion about the effectiveness of interventions. Analytic Products: Graphical displays of the data and a table that displays the key elements of each study. Final product: A mathematic score that represents the strength of the effect of an intervention or the relationships between two variables. Identification of variables that moderate or mediate the effects or relationships.
QUALITATIVE **Meta-Study**	**What is it?** “Meta-study is a research approach involving analysis of the theory, methods, and findings of qualitative research and the synthesis of these insights into new ways of thinking about phenomenon” [[54], p.1]. **Data type:** Three analytic components are undertaken prior to synthesis. Data includes qualitative findings (meta-data), research methods (meta-method), and/or philosophical/theoretical perspectives (meta-theory). **Research question:** A relevant, well-defined research question is used. **Critical appraisal:** According to Paterson et al. [54], primary articles are appraised according to specific criteria; however the specific appraisal will depend on the requirements of the meta-study. Studies of poor quality will be excluded. Data from included studies may also be excluded if reported themes are not supported by the presented data.	Analysis of research findings, methods, and theory across qualitative studies are compared and contrasted to create a new interpretation [53].	Paterson et al. [54] propose a clear set of techniques: Choose an analytic approach (e.g. grounded theory, thematic analysis). Use specific sampling techniques according to inclusion/exclusion criteria, including searching for disconfirming cases that challenge the emerging theory. Regardless of approach, group studies according to characteristics (e.g., disease) and treat each group as a case [49] Engage in three distinct types of analysis, i.e. meta-data, meta-study, meta-theory (may be undertaken concurrently). Synthesize analysis into a theory.	Through the three meta-study processes, researchers create a “meta-synthesis” which brings together ideas to develop a mid-range theory as the product.
QUALITATIVE **Meta-Ethnography**	**What is it?** Meta-ethnography entails choosing relevant empirical studies to synthesize through repetitive reading while noting metaphors [61]–[62]. Noblit and Hare explain that “metaphors” refer to “themes, perspectives, organizers, and/or concepts revealed by qualitative studies” [[61], p.15]. These metaphors are then used as data for the synthesis through (at least) one of three strategies including reciprocal translation, refutational synthesis, and/or line of argument syntheses. A meta-ethnographic synthesis is the creation of interpretive (abstract) explanations that are essentially metaphoric. The goal is to create, in a reduced form, a representation of the abstraction through metaphor, all the while preserving the relationships between concepts [61]. **Data type:** Qualitative research studies and findings on a specific topic. **Research question:** An “intellectual interest” [[61], p.26] begins the process. Then, a relevant research question, aim, or purpose is developed. **Quality appraisal:** Researchers are divided on the merits of critical appraisal and whether or not it should be a standard element in meta-ethnography [60]. Some researchers choose to follow pre-determined criteria based on critical appraisal [e.g., [62]], whereas others do not critically appraise.	To synthesize qualitative studies through a building of “comparative understanding” [[61], p.22] so that the result is greater than the sum of the parts. Noblit and Hare summarize that meta-ethnography is “a form of synthesis for ethnographic or other interpretive studies. It enables us to talk to each other about our studies; to communicate to policy makers, concerned citizens, and scholars what interpretive research reveals; and to reflect on our collective craft and the place of our own studies within it” [[61], p.14].	Methods used in meta-ethnography generally following the following: Frame the study broadly by an interest, aim or purpose and ultimately, a research question. Create inclusion/exclusion criteria. Conduct a review of the literature based on who the audience will be, what is credible to the audience, what accounts are available, and what the researchers' interests are in the study [61]. Identify all the appropriate studies in a field through repeated readings. Noblit and Hare [61] identified three possible analysis strategies (all do not have to be completed): Reciprocal translational analysis. Key themes, metaphors, or concepts are identified and translated into each other to create the most representative concept. Refutational synthesis. Contradictions between key themes, metaphors, or concepts are examined and explained. Lines of argument synthesis. Interpretation is created from comparison of findings across distinct studies.	The product of a meta-ethnography is a mid-range theory that has greater explanatory power than could be otherwise achieved in a conventional literature review.
QUALITATIVE **Grounded Formal Theory (GFT)**	**What is it?** A grounded formal theory (GFT) is a synthesis of substantive grounded theories (GTs) to produce a higher order, more abstract theory that goes beyond the specifics of the original theories. GFT takes into account the conditions under which the primary study data were collected and analyzed to develop a more generalized and abstract model [31]. **Data type:** Substantive GTs were originally constructed using the methodology developed by Glaser & Strauss [86]. While some synthesis approaches emphasize including all possible primary GT studies, the concept of saturation in GFT (see Methods column) allows limiting the number of reviewed papers to emphasize robustness rather than completeness [50]. **Research question:** GFT begins with a phenomenon of focus [51]. Analytic questions and the overall research question emerge throughout the process. **Quality appraisal:** There is no discussion in the GFT literature about critically appraising the studies to be included. However, the nature of the analytic process suggests that critical appraisal may not be relevant. The authenticity and accuracy of data in a GFT are not an issue because, for the purposes of generating theory, what is important is the conceptual category and not the accuracy of the evidence. The constant comparative method of GFT will correct for such inaccuracies because each concept must “earn” its way into the theory by repeatedly showing up [67]–[68].	The intent of GFT is to expand the applicability of individual GTs by synthesizing the findings to provide a broad meaning that is based in data and is applicable to people who experience a common phenomenon across populations and context [51]. The focus is on the conditions under which theoretical generalizations apply. GFT aims “to bring cultural and individual differences into dialogue with each other by seeking a metaphor through which those differences can be understood by others” [[31], p.1354].	GFT uses the same methods that were used to create the original GTs in the synthesis [48],[51]. Specific elements of the analytic process include: Theoretical sampling - sample size is determined through purposive and theoretical sampling strategies to answer emerging questions [37],[51]. Constant comparative analysis -the analyst identifies concepts and their relationship with other data, and compares theoretical ideas to prior and subsequent data. Memoing - documentation of hunches, decisions, and modifications during analysis. Saturation - the point at which continued data collection and analysis brings only repeated concepts or ideas. Coding - begins at a descriptive level and progresses towards a more abstract and theoretical level. Findings are synthesized and translated across studies.	A GFT is a mid-range GT that has “fit, work and grab”: that is, it fits the data (concepts and categories from primary studies), works to explain the phenomenon under review, and resonates with the readers' experiences and understandings. Thorne et al. suggest that a GFT is “an artistic explanation that works for now, a model created on the basis of limited materials and a specific, situated perspective within known and unconscious limits of representation” [[31], p.1354].
QUALITATIVE **Concept Analysis**	**What is it?** Concept analysis is a systematic procedure to extract attributes of a concept from literature, definitions and case examples to delineate the meaning of that concept with respect to a certain domain or context. **Data type:** Most writings on concept analysis do not specify the data type. However, our scan of the methodological and empirical literature on concept analysis suggests that although the analytic approach in concept analysis is qualitative, quantitative study designs and data can be used to address the questions related to defining the meaning of a concept [e.g. [99], [141]–[142]]. **Research question:** Requires the researcher to isolate or identify a conceptual question or concept of interest. **Quality appraisal:** Quality appraisal is not typically attended to in concept analyses. Rather, researchers are interested in all instances of actual use of a concept (or surrogate terms) [142].	Concept analysis is used to extend the theoretical meaning of a concept or to understand a conceptual practice problem [142]–[143]. In this case, concepts are cognitive descriptive meanings utilized for theoretical or practical purposes. Concept analysis is used to identify, clarify, and refine or define the meaning of a concept and can be used as a first step in theory development [47],[144].	There are varied procedural techniques attributed to various authors such as Wilson [98], Walker & Avant [45], Chinn & (Jacobs) Kramer [145]–[146], Rodgers & Knafl, [46], Rodgers [99], Schwartz-Barcott & Kim [147], and Morse [47]. Despite varied techniques, steps generally include: Determine the purpose and aims. Delineate domains or boundaries of the concept. Draw on literature, dictionary meanings and/or cases. Analyze data sources to determine qualifying attributes. Develop a prototype case and compare against contrary or borderline cases. Test the practical significance. Formulate defining features. Relate to theoretical importance or practice application [46],[141],[148].	Concept analysis generates a definition of a concept that may be used to operationalize phenomena for further research study [143] or theory development [144].
EMERGING **Scoping Review**	**What is it?** Although no universal definition exists, there are some common elements of scoping reviews [129],[149]. They are exploratory projects that systematically map the literature on a topic, identifying the key concepts, theories, sources of evidence, and gaps in the research. It involves systematically selecting, collecting and summarizing knowledge in a broad area [130]. A scoping review is used to address broad topics where many different study designs and methods might be applicable. It may be conducted as part of an ongoing review, or as a stand-alone summary of research. Whereas a systematic review assesses a narrow range of quality-assessed studies to synthesize or aggregate findings, a scoping review assesses a much broader range of literature with a wide focus and does not synthesize or aggregate the findings [59]. **Data type:** Includes studies using any data type or method. May include empirical, theoretical or conceptual papers. Exclusion and inclusion criteria are inductively derived and based on relevance rather than on the quality of the primary studies or articles [150]. **Research question:** The question is stated broadly and often becomes refined as the study progresses. One or more general questions may guide the review. **Quality appraisal:** The scoping review does not provide an appraisal of the quality of the evidence. It presents the existing literature without weighting the evidence in relation to specific interventions.	The purpose of a scoping review is to examine the extent, range and nature of research activity in an area. It is done to identify where there is sufficient evidence to conduct a full synthesis or to determine that insufficient evidence exists and additional primary research is needed [130],[151]. It may be done for the purpose of disseminating research findings [63] or to clarify working definitions and the conceptual boundaries of a topic area [129].	Arksey and O'Malley [63] recommend a 5 step process for conducting a scoping review: Identification of a broad research question. Identification of relevant studies covering a wide breadth of literature and a variety of sources via databases, reference lists, and hand-searching key journals. This process may include consultation with key stakeholders. Inclusion and exclusion criteria are identified as the review progresses. The data are sifted, sorted, compared and contrasted according to key issues and themes. Data are charted to allow for comparison and to ensure a uniform approach. Finally, the information is summarized and reported. Clear documentation of the methodology is important so that the reader can determine any potential reporting bias. More recently, Levac et al. [129] have proposed recommendations to clarify and enhance each stage of the framework described above.	The product of a scoping review will depend on the purpose for which it is conducted. In general, however, the narrative report provides an overview of all reviewed material. The product generally includes: Basic numerical or narrative analysis of the extent, nature and distribution of the studies included with tables, graphs, and charts. Thematic organization of the literature (e.g., by intervention type, or by competing theoretical perspectives). Summary statement about what is known and not known (e.g., in the literature).
EMERGING **Rapid Review**	**What is it?** Rapid review of the literature provides a quick, rather than comprehensive, overview of the literature on a narrowly defined issue. Rapid review evolved out of a need to inform policy makers about issues and interventions in a timely manner [152]. It is often proposed as an intermediary step to be followed by a more comprehensive review. **Data type:** The literature is often narrowly defined, focusing on a specific issue or a specific local, regional, or federal context [152]. It can include diverse study designs, methods, and data types as well as peer reviewed and gray literature. **Research question:** Rapid reviews require a thorough understanding of the intended audience and a specific, focused research question. **Quality appraisal:** Rapid reviews typically do not include an assessment of the quality of the literature, nor do they always include the views of experts and/or reviews by peers [152].	The purpose is to produce a fast review of the literature, within a defined and usually limited time frame, on a question of immediate importance to a stakeholder group.	There is no standardized methodology as yet, but the depth and breadth of the review depends upon the specific purpose and the allotted time frame. Rapid reviews typically take one to nine months. They begin with a needs assessment followed by formulation of a purpose statement and research question, definition of the context, and review of the literature [152]–[154]. A review of the literature is streamlined in numerous ways including: Accessing only published or online literature; Limiting by publication date, the number of databases, or language; Searching electronic journals only; Narrowing to specific geographic settings or contexts; Restricting the timeframe during which articles are assessed; Limiting contact with authors/industry or key stakeholders for clarification, follow-up, or input [152]–[154]. References are retrieved, selected, summarized or synthesized, and a report is created. The public may be consulted about the results [152]. It is important that those conducting a rapid review describe the methodology in detail to promote transparency, support transferability, and avoid misrepresenting the veracity of the findings [152].	Typically a concise report is written for macro-level decision-makers that answer the specific review question.
EMERGING **Meta-Narrative Synthesis (MNS)**	**What is it?** MNS is a new form of systematic review that addresses the issues of synthesizing a large and complex body of data from diverse and heterogeneous sources. At the same time, it is systematic in that it is conducted “according to an explicit, rigorous and transparent method” [[67], p.418]. The approach moves from logico-scientific reasoning (which underlies many approaches to synthesis) to narrative-interpretive reasoning. The unit of analysis for the synthesis is the unfolding “storyline” of a research tradition over time. Five key principles underlie the methodology: pragmatism, pluralism, historicity, contestation, and peer review. **Data type:** This methodology involves the judicious combination of qualitative and quantitative evidence, and the theoretical and empirical literature. **Research question:** The original research question is outlined in a broad, open-ended format, and may shift and change through the process. **Quality appraisal:** MNS uses the criteria of the research tradition of the primary study to judge the quality of the research, generally as set out in key sources within that tradition.	The purpose is to summarize, synthesize and interpret a diverse body of literature from multiple traditions that use different methods, theoretical perspectives, and data types.	The steps to conduct a MNS [67],[84]–[85] include the following: Planning Phase: Assemble a multidisciplinary team, outline an initial broad question, and agree on outputs. Search Phase: Initially search by intuition, informal networking, browsing to map diversity of perspectives. Search for seminal papers. Search for empirical papers in databases, hand searching key journals, and snowballing. Mapping Phase: For each research tradition, identify key elements of the research paradigm, key actors and events in unfolding traditions, and prevailing language/imagery. Appraisal Phase: Evaluate each study for validity/relevance, extract and collate key results, group comparable studies. Synthesis Phase: Identify all key dimensions of the problem/issue, provide a narrative account of each contribution, treat conflicting findings as higher order data and explain in terms of contestation between different paradigms from the original data. Recommendations Phase: Summarize overall messages and relevant evidence; distil and discuss recommendations for policy, practice, and research.	The product of a MNS is: A set of meta-narratives illustrating the story lines of various research traditions related to a common area or question; An overarching conceptual framework that explains the phenomenon of interest.
EMERGING **Realist Synthesis**	**What is it?** A realist synthesis is a review of complex social interventions and programs that seek to unpack the mechanisms by which complex programs produce outcomes, and the context in which the relationship occurs. This is in contrast to systematic reviews, which aim to synthesize studies on whether interventions are effective. Realist synthesis seeks to answer the question: What works for whom, in what ways and under what circumstances? This form of synthesis represents a review logic not a review technique [69]. Instead of a replicable method that follows rigid rules, the logic of realist review is based on principles. It reflects a shift away from an ontology of empirical realism to one of critical realism [155]. **Data type:** There is no specific data preference but will include quantitative, qualitative and grey literature. Because the focus is on the mechanisms of action and their context, seemingly disparate bodies of literature and diverse methodologies are included. The focus is upon literature that emphasizes process with detailed descriptions of the interventions and context. **Research question:** The review question is carefully articulated, prioritizing different aspects of an intervention [69]. It can be a broad question. **Quality appraisal:** Realist review supports the principle that high quality evidence should be used but takes a different position than in systematic reviews on how the evidence is to be judged. It rejects a hierarchical approach to quality because multiple methods are needed to identify all aspects of the context, mechanisms and outcomes. Appraisal checklists are viewed skeptically because they cannot be applied evenly across the diverse study types and methods being reviewed. Thus, quality appraisal is seen as occurring in stages with a focus on the relevance of the study or article to the theory under consideration, and the extent to which an inference drawn has sufficient weight to make a credible contribution to the test of a particular intervention theory [69].	The purpose of a realist synthesis is to guide program and policy development by providing decision makers with a set of program theories that identify potential policy levers for change. Within its explanatory intent, there are four general purposes: Reviewing for program theory integrity. Reviewing to adjudicate between rival program theories. Reviewing the same theory in different settings or with different populations. Reviewing official expectations against actual practice [see [69],[107]].	Pawson et al. [69] identify 5 steps: Clarify scope: Identify the review question, nature of the intervention, circumstances for its use, and policy objectives; Refine the purpose of the review; Make explicit the program theory or theories (e.g., the underlying assumptions about how the intervention is meant to work), synthesize theories, and design a theoretical framework. Search for evidence: Conduct an exploratory search; Identify key program theories and refine inclusion criteria; Purposively sample to test a subset of theories, with additional snowball sampling; Search for new studies when review is almost completed. Appraise primary studies and extract data: Use judgment to supplement critical appraisal checklists; Develop data extraction forms; Extract data. Synthesize evidence and draw conclusions: Synthesize data to refine program theory; Let the purpose of the review lead the synthesis process; Use contradictory evidence to create insights about the impact of context; Present conclusions as a set of decision points. Disseminate, implement and evaluate: Draft and test recommendations with key stakeholders focusing on what may influence policy; Work with policy makers and practitioners to apply recommendations; Evaluate the extent to which recommendations lead to program adjustments.	Pawson [68] explains that realist synthesis ends up with useful, middle-range theory. However, the product of a realist review combines theoretical understanding with empirical evidence. It focuses on explaining the relationships among the context in which an intervention takes place, the mechanisms by which it works, and the outcomes produced [68]–[69]. Recommendations for dissemination and implementation are explicitly articulated. The result is a series of contextualized decision points that describe the contingencies of effectiveness. That is, a realist review provides an explanatory analysis that answers the original question of “what works for whom, in what circumstances, in what respects, and how” [[69], p.21].
EMERGING **Critical Interpretive Synthesis (CIS)**	**What is it?** CIS is a methodology with an explicit orientation to theory generation, developed to respond to the need identified in the literature for rigorous methods to synthesize diverse types of research evidence generated by diverse methodologies [71] particularly when the body of evidence is very complex [72]. Thus, it was developed to address the limitations of conventional systematic review techniques. It involves an iterative process and recognizes the need for flexibility and reflexivity. It addresses the criticism that many approaches to syntheses are insufficiently critical and do not question the epistemological and normative assumptions reflected in the literature [72]. CIS is “sensitized to the kinds of processes involved in a conventional systematic review while drawing on a distinctively qualitative tradition of inquiry” [[72], p.35]. **Data type:** CIS utilizes data from quantitative and qualitative empirical studies, conceptual and theoretical papers, reviews and commentaries. **Research question:** It is neither possible nor desirable to specify a precise review question in advance. Rather the process is highly iterative and may not be finalized until the end of the review.**Quality appraisal:** There is no hierarchy of designs for determining the quality of qualitative studies and, furthermore, no consensus exists on whether qualitative studies should even be assessed for quality [72]. Studies for inclusion are not selected on the basis of study design or methodological quality. Rather, papers that are relevant are prioritized. However, papers that are determined to be fatally flawed are excluded on the basis of a set of questions for determining quality [see [71]]. Often, however, judgments about quality are deferred until the synthesis phase because even methodologically weak papers can provide important theoretical or conceptual insights [73].	The purpose of CIS is to develop an in-depth understanding of an issue/research question “by drawing on broadly relevant literature to develop concepts and theories that integrate those concepts” [[73], p.71]. The overarching aim is to generate theory.	The developers of CIS explicitly reject a staged approach to the review. Rather, the processes are iterative, interactive, dynamic and recursive. It includes these general categories of activities [71]–[72]: Formulate the research question: The question is not formulated in advance because the aim is to allow the definition of the phenomenon of interest to emerge from analysis. Search the literature: Involves an organic approach using multiple search strategies (e.g., websites, reference chaining, contacting experts) in addition to a more structured approach; Draw on the expertise of the team to identify relevant studies; Identify relevant papers that can form a sampling frame. Sample: May be selective and purposive, with emergent and flexible inclusion criteria; Ongoing selection is guided by theoretical sampling based on the emerging conceptual framework. Determination of quality: See “quality appraisal” section. Data extraction: Forms to guide this process can be useful, but with a huge database may be practically impossible; An informal process (highlighting text) can prove helpful. Interpretive synthesis: Synthesis is based, in part, on the meta-ethnography strategies of reciprocal translational analysis, refutational synthesis, and lines of argument synthesis, but the authors greatly modified these to accommodate the diversity of literature (meta-ethnography used purely qualitative studies); The aim of the analysis is to produce a synthesizing argument, beginning with a detailed inspection of papers, gradually identifying recurring themes and developing a critique, constantly comparing concepts developed against the data and identifying the relationships among them.	The product is a “synthesizing argument” that “links existing constructions from the findings to ‘synthetic constructs' (new constructs generated through synthesis)” [[73], p.71]. The synthesizing argument integrates evidence from across the studies in the review into a coherent theoretical framework [71]–[72]. This may be represented as a “conceptual map” that identifies the main synthetic constructs and illustrates the relationships among them [73].
